# Comparative Analysis of Inflammatory and Heavy Metal Biomarkers in Exclusive E-Cigarette Users, Combustible Tobacco Users, and Non-Users Aged 18–30: A Cross-Sectional NHANES Analysis

**DOI:** 10.3390/jox15020053

**Published:** 2025-04-01

**Authors:** Luke Manietta, William Drake, Wasantha Jayawardene

**Affiliations:** 1 SIU School of Medicine, Springfield, IL 62702, USA; 2 Department of Public Health, School of Human Sciences, Southern Illinois University—Carbondale, Carbondale, IL 62901, USA

**Keywords:** e-cigarettes, cigarettes, inflammatory biomarkers, heavy metals, NHANES

## Abstract

While cigarette smoking has declined, e-cigarette use among young adults has increased in the USA. This cross-sectional study compared complete blood counts, C-reactive protein, and select blood metals (lead, cadmium, mercury, selenium, manganese) among exclusive combustible tobacco users, exclusive e-cigarette users, and non-users using NHANES data from 2013–2023 in the USA. The goal of this study was to compare biomarker data from e-cigarette users and combustible tobacco users to that of non-users. Among 756 participants aged 18–30, 229 reported no tobacco use, 74 used only e-cigarettes, and 453 smoked only combustible tobacco. Survey-weighted analyses adjusted for age, sex, race/ethnicity, and body mass index revealed that exclusive combustible use was associated with significantly elevated white blood cell counts, hemoglobin, hematocrit, mean corpuscular volume, lymphocytes, monocytes, neutrophils, and higher blood lead and cadmium. E-cigarette-only use showed fewer deviations overall but included higher mean corpuscular hemoglobin concentration and lower cadmium, selenium, and methyl mercury relative to non-users. Neither group differed significantly in red blood cell count or C-reactive protein, and inorganic/ethyl mercury and manganese levels were largely unchanged. These findings underscore pronounced hematologic and metal-related alterations in combustible tobacco users and fewer, but not negligible, changes among e-cigarette users. Further research is needed to determine the long-term health implications of exclusive e-cigarette use, including potential impacts on antioxidant micronutrient levels.

## 1. Introduction

Despite the decline in tobacco use prevalence in the United States, its popularity remains a significant public health risk. As of 2022, 19.8% of U.S. adults used tobacco products with cigarettes being the most common form of consumption, followed by e-cigarettes, smokeless tobacco, and cigars [1]. However, the popularity of e-cigarettes is on the rise among young adults aged 18–24 [2,3]. Additionally, this age group has been heavily targeted by e-cigarette marketing [4]. E-cigarettes are an arguably safer alternative to combustible tobacco and referred to as healthier and safer than traditional tobacco products on social media [5,6]. However, most young e-cigarette users have no history of cigarette use. A total of 65.5% of those aged 18–24, and 21.6% aged 24–44 have never used combustible tobacco, which emphasizes concerns about e-cigarettes’ role in initiating nicotine dependence [1,2,7]

The age of first tobacco use is a critical determinant of long-term use [8]. Individuals who begin smoking before the age of 21 are more likely to become dependent on nicotine and have lower odds of attempting and intending to quit [9,10]. Despite combustible cigarettes’ declining popularity among young adults ages 18–24, 5.4% of this group still smokes [11]. On the other hand, this same population represents the age group where e-cigarettes are gaining the most popularity as a combustible tobacco substitute [12]. However, e-cigarettes are not seeing the same rise across age groups. In groups of adults 30 and older e-cigarettes have only seen an increase in use among former users, not first-time users [5,13]. Given these shifting trends, individuals aged 18–30 represent a key demographic for studying the impacts of tobacco and e-cigarette use.

The health impacts of cigarettes are well-documented. The effect cigarettes have on white blood cell counts is well understood, including increased counts of leukocytes, neutrophils, monocytes, and increased hemoglobin [14]. Some have also found a direct relationship between combustible cigarette use and an increase in serum C-reactive protein (CRP) levels [15]. However, this relationship is dose-dependent, with heavier users seeing greater increases, and this can be influenced by other factors including sex [16,17]. Additionally, cigarette use is associated with increased serum concentrations of lead and cadmium [18]. It is believed that the use of e-cigarettes lowers the risk of exposure to heavy metals, compared to combustible tobacco [19,20]. E-cigarettes also may increase circulating levels of CRP [21]. Overall, the health impacts of e-cigarette use are poorly understood due to insufficient evidence [22]. Further investigation into these metrics is imperative for the growing population that solely use e-cigarettes [23].

The primary objective of this study is to compare complete blood count (CBC) values, serum C-Reactive Protein (CRP) levels, and serum levels of key heavy metals across three groups: people that only smoke combustible tobacco products (cigarettes, cigars, pipes, and/or hookah), e-cigarette only users, and those who do not use tobacco or nicotine-containing products, including e-cigarettes. The secondary objective is to assess the magnitude and significance of the differences across these three groups. These assessments were made based on NHANES data, controlling for confounding variables including sex, race/ethnicity, BMI, and alcohol use. It is hypothesized that e-cigarette use will increase WBC count, inflammatory markers (CRP), and serum toxic-metal levels when compared to non-users, though not as significantly as combustible tobacco users.

## 2. Materials and Methods

### 2.1. Study Design and Data Source

This cross-sectional study used data from the National Health and Nutrition Examination Survey (NHANES), a nationally representative survey conducted by the National Center for Health Statistics (NCHS) of the Centers for Disease Control and Prevention (CDC). NHANES employs a complex, multistage probability sample design to collect detailed health and nutrition information on noninstitutionalized U.S. residents [24]. Data were drawn from combined survey cycles covering 2013–2023, after merging multiple NHANES component files (for example, demographic, dietary, laboratory, and examination data). All participants provide written informed consent, and study protocols receive annual approval from the NCHS Research Ethics Review Board. The public-use NHANES data files are de-identified and available on the NHANES website.

### 2.2. Participants

Among individuals of all ages in NHANES, the sample was restricted to participants between the ages of 18 and 30. The analysis specifically targets adults aged 18–30, a demographic in which e-cigarette uptake has risen most rapidly and is less confounded by chronic diseases or comorbidities commonly seen in older adults. Focusing on younger adults helps isolate the acute and subacute effects of different nicotine products on biomarkers without the complicating factor of long-term illness. Participants missing key variables for tobacco use classification and those missing critical weights or design variables were excluded. Individuals with zero or invalid sample weights were also excluded. Additionally, those who had indicated a current or previous liver condition were excluded.

### 2.3. Tobacco Use Classification

Tobacco use status was classified into three mutually exclusive groups—Combustible Only, E-Cigs Only, or No Tobacco—using information on recent product use, lifetime cigarette history, and second-hand smoke exposure. First, a specific indicator was created to identify participants who had used combustible tobacco products in the past five days, which included cigarettes, cigars, pipes, and hookah. Anyone who met these criteria was assigned to the Combustible Only group. Next, respondents were coded as E-Cigs Only if they reported e-cigarette use within the last five days, had never smoked 100 cigarettes in their lifetime, did not currently smoke cigarettes, had no reported use of other tobacco products in the past five days, and had no secondhand smoke exposure in their primary residence. Finally, those who did not currently smoke, had no e-cigarette use or other tobacco use in the past five days, and did not have second-hand smoke exposure in their primary residence were categorized as No Tobacco. Anyone who failed to meet these specifications and those who had conflicting data were excluded to maintain clear, non-overlapping categories.

NHANES’ five-day timeframe for self-reported tobacco use was used to define “current” e-cigarette or combustible use, recognizing that this interval may overlook intermittent or weekend-only users. Similarly, dual users who did not report both products within the same window remained undetected. Nevertheless, this was the only consistent measure available across all survey cycles, enabling the identification of exclusive usage. Consequently, individuals indicating both e-cigarettes and combustible products during the five-day period were excluded, which reduces potential confounding but does not capture the reality of dual use in many smokers. In addition, NHANES does not uniformly track daily consumption (e.g., pack-years, vaping frequency), limiting the ability to stratify by intensity or dose. While this simpler classification streamlines our comparisons, it represents a trade-off that is addressed further in the study’s limitations.

It is important to note that our final sample proportion of combustible or e-cigarette users does not reflect nationwide prevalence. Rather, our classification aims to isolate exclusive recent usage categories (i.e., no overlapping forms of tobacco or secondhand smoke). This approach inflates the percentage of “exclusive” users compared to national averages, but allows for a cleaner comparison of biomarker outcomes across distinct tobacco user groups.

### 2.4. Laboratory Measures

Data were extracted for laboratory markers associated with inflammation and immune function, including complete blood count (CBC) measures (white blood cell count [WBC], hemoglobin, hematocrit, and RBC indices) and high-sensitivity C-reactive protein (CRP). Blood metals of interest included lead, cadmium, total mercury, selenium, manganese, methylmercury, ethylmercury, and inorganic mercury. Arsenic, nickel, cobalt, chromium, and other metal levels were unable to be included due to the NHANES survey only testing these variables in individuals over the age of 30. NHANES conducts these laboratory analyses in a standardized mobile examination center (MEC) using validated assays [24]. The relevant lower limit of detection (LOD) is reported for each metal, and half-LOD values are assigned for measurements below detection, consistent with CDC guidelines [24].

### 2.5. Covariates

Sociodemographic and health variables included age (continuous, restricted to <40 years), gender (male or female, self-reported), and race/ethnicity (Mexican American, Other Hispanic, Non-Hispanic White, Non-Hispanic Black, Non-Hispanic Asian, or Other/Multiracial). Body mass index (BMI), as measured in the MEC, was also included. Additionally, diabetes status was also considered as a covariate, but was not included in the final analysis due to the low number of individuals with diabetes included.

While certain socioeconomic or occupational factors can influence tobacco use and metal exposure, many were incompletely reported for this age range in NHANES. Including them would have significantly reduced the sample size and compromised analytical power. Consequently, the final models focus on the core demographic measures listed above, acknowledging the possibility of residual confounding.

### 2.6. Statistical Analysis

All analyses incorporated the NHANES sample weights to account for the complex, multistage design and to produce nationally representative estimates. The “survey” package (version 4.2-2) in R (version 4.3.1) was used to specify the appropriate design variables (masked variance pseudo-strata, pseudo-PSU, and the combined multi-year MEC exam weight (WTMEC2YR) [25,26]. Descriptive statistics included unweighted counts of participants in each tobacco group. Weighted means (±standard errors) for continuous variables were calculated and weighted proportions were estimated for categorical covariates.

Comparisons of laboratory markers by tobacco group included weighted means (with standard errors) of WBC, RBC indices, platelets, and CRP across the three categories (No Tobacco, E-Cigarettes Only, and Combustible Only). Weighted means (with standard errors) were also estimated for lead, cadmium, mercury, selenium, and manganese. Certain measures were log-transformed before regression analyses to address right-skewed distributions. Survey-weighted generalized linear models were then used to regress each biomarker on tobacco group (with No Tobacco as the reference) while adjusting for age, gender, race/ethnicity, and BMI. Beta coefficients (or exponentiated coefficients for log-transformed outcomes) and 95% confidence intervals (CI) were reported. Hypothesis testing used adjusted Wald F-tests in accordance with NHANES survey design, and two-sided *p*-values < 0.05 were considered statistically significant. Sample sizes in each analytic step varied due to missing data for certain biomarkers.

## 3. Results

A total of 756 adults (unweighted) met the inclusion criteria: 229 (30.3%) reported no tobacco use, 74 (9.8%) used only e-cigarettes, and 453 (59.9%) smoked only combustible tobacco products (Table 1). On average, the e-cigarette group was the youngest (22.21 ± 0.41 years), whereas participants without tobacco use had the highest mean age (25.01 ± 0.20 years), and those who use combustible tobacco fell in between (23.67 ± 0.17 years). Mean BMI was greatest among the no-tobacco group (27.98 ± 0.58 kg/m^2^), followed by combustible tobacco users (27.10 ± 0.36 kg/m^2^) and then e-cigarette users (26.89 ± 1.52 kg/m^2^). Men comprised 60.9% of the no-tobacco group, 57.5% of combustible tobacco users, and 52.2% of e-cigarette users. Non-Hispanic White adults represented roughly half or more of each group, ranging from 48.2% among e-cigarette users to 63.3% among those with no tobacco use.

Table 2 presents the weighted mean values for complete blood counts (CBC) and selected metals stratified by tobacco status. Overall, combustible-only participants (*n* = 430) again showed the highest WBC counts (8.03 ± 0.15 × 10^3/^µL) compared with those reporting no tobacco (n = 215; 7.42 ± 0.17 × 10^3^/µL) and e-cigarette-only users (n = 68; 7.12 ± 0.32 × 10^3^/µL). Red blood cell indices, such as RBC count, hemoglobin, and hematocrit, were also greatest in the combustible group (e.g., hemoglobin = 14.59 ± 0.09 g/dL), while the no-tobacco and e-cigarette groups showed slightly lower values. Platelet counts, though relatively similar across groups, were modestly lower in the no-tobacco (244.22 ± 4.39 × 10^3^/µL) and e-cigarette (244.42 ± 9.65 × 10^3^/µL) categories than among combustible participants (246.93 ± 3.88 × 10^3^/µL).

Regarding metals, blood cadmium was markedly elevated among combustible-only users (0.75 ± 0.05 µg/L) versus no-tobacco (0.27 ± 0.02 µg/L) and e-cigarette-only participants (0.14 ± 0.009 µg/L). Blood lead followed a similar trend, highest in the combustible group (0.91 ± 0.05 µg/dL), moderate among no-tobacco (0.77 ± 0.04 µg/dL), and lowest for e-cigarette users (0.66 ± 0.13 µg/dL). In contrast, total mercury levels were highest in the no-tobacco group (1.15 ± 0.26 µg/L) and lowest for e-cigarette users (0.61 ± 0.13 µg/L). This pattern remained for methyl mercury, which was most elevated among no-tobacco participants (1.04 ± 0.25 µg/dL) and lowest in e-cigarette users (0.51 ± 0.13 µg/dL). Finally, CRP values showed the highest mean in the combustible group (3.45 ± 0.58 mg/L), moderate in no-tobacco participants (2.97 ± 0.54 mg/L), and lowest among the e-cigarette subset (2.10 ± 0.50 mg/L).

In the adjusted survey-weighted regression analyses (Table 3), combustible-only use was associated with several notable alterations compared to no-tobacco use. In particular, the combustible group displayed higher white blood cell counts (β = 0.83, *p* < 0.001), hemoglobin (β = 0.42, *p* = 0.003), hematocrit (β = 1.26, *p* = 0.002), mean corpuscular volume (β = 1.24, *p* = 0.012), lymphocyte number (β = 0.15, *p* = 0.048), monocyte number (β = 0.04, *p* = 0.011), and neutrophil number (β = 0.62, *p* < 0.001) relative to non-users. In contrast, e-cigarette-only use was characterized by a higher mean corpuscular hemoglobin concentration (β = 0.30, *p* = 0.012) but did not differ significantly in other CBC or inflammatory markers. Notably, RBC count and C-reactive protein were not significantly different from non-users in either user group.

Analysis of heavy metals (Table 4) confirmed substantially elevated blood lead and cadmium among combustible-only users, aligning with known constituents in combustible tobacco products. Specifically, the combustible group showed significantly higher blood lead (β = 0.18, *p* = 0.005) and markedly increased blood cadmium (β = 0.95, *p* < 0.001) compared to non-users. By contrast, e-cigarette-only use was associated with significantly lower cadmium (β = −0.51, *p* = 0.001) and a modest yet significant reduction in blood selenium (β = −0.08, *p* = 0.001). Lead levels did not differ significantly in the e-cigarette group, compared to non-users (*p* = 0.265). Neither tobacco group exhibited notable differences in blood manganese or inorganic/ethyl mercury, compared to non-users. However, e-cigarette-only users showed significantly lower methyl mercury (β = −0.39, *p* = 0.049), while the change observed in the combustible group (*p* = 0.424) was not statistically significant.

## 4. Discussion

A central finding of this study is that combustible-only tobacco use is associated with a broad range of hematologic alterations, while e-cigarette-only use has fewer and more modest effects. Specifically, those who use combustible tobacco exhibited significantly higher WBC counts, hemoglobin, hematocrit, mean corpuscular volume, and certain white blood cell subtypes (lymphocytes, monocytes, neutrophils) relative to non-users. This observation strongly supports prior evidence that combustible tobacco products drive an inflammatory response, potentially triggered by oxidative stress, endothelial dysfunction, and repeated exposure to toxic combustion byproducts [14]. Elevated neutrophils and monocytes, in particular, reinforce the notion that chronic, low-grade immune activation is a hallmark of cigarette smoking. Another plausible explanation for increased hematocrit is the mild hypoxic environment created by cigarette smoke, which may stimulate the body to produce more red blood cells to compensate.

By contrast, the e-cigarette-only group displayed fewer significant deviations from non-users in classic inflammatory or RBC measures. The single notable finding was a higher mean corpuscular hemoglobin concentration (MCHC), whereas WBC counts and CRP remained largely unaffected. The findings suggest that users of e-cigarettes appear to exhibit a significantly lower inflammatory response than those who smoke combustible products. E-cigarette liquids still contain nicotine, flavoring chemicals, and other solvents (e.g., propylene glycol, glycerin), all of which may pose as-yet-underrecognized health risks over longer durations or with different puffing patterns. However, it should be noted that e-cigarettes remain a healthier alternative to combustible tobacco [6]. Indeed, conflicting research exists regarding the cardiopulmonary impacts of e-cigarettes, warranting ongoing, long-term studies [27,28].

Turning to heavy metals, the data confirm markedly higher cadmium among those who smoke combustible tobacco, consistent with the well-established presence of cadmium in tobacco leaves and its efficient absorption during combustion [18]. Lead exposure was also significantly elevated in the combustible group, paralleling prior findings that cigarette smoke can serve as a notable lead source. Taken together, these data for metals underscore an important advantage of switching completely from combustible products to alternatives, in that e-cigarette users exhibited much lower cadmium and did not significantly differ in blood lead levels compared to non-users. This has been attributed to minimal transfer of cadmium to e-cigarette aerosol compared to cigarette smoke [29]. The current results for e-cigarette users are consistent with the current literature which indicates that heavy metal exposure in e-cigarette users is lower in comparison to those who use cigarettes [30].

Interestingly, e-cigarette-only participants also showed reduced blood selenium and lower methyl mercury relative to non-users. Selenium is an essential trace element with antioxidant properties, and variations in its concentration could reflect differences in diet, absorption, or metabolic demands among e-cigarette users. There have been a few studies that have mentioned the observation of decreased selenium concentrations in those who use combustible cigarettes [31]. However, it remains to be seen how these findings translate to e-cigarette usage and whether this is due to nicotine itself or another chemical. Meanwhile, the finding of lower methyl mercury levels in e-cigarette users, though statistically significant, remains mechanistically speculative; it may reflect differences in lifestyle or dietary preferences rather than any direct effect of vaping.

Neither tobacco group showed significant changes in manganese or inorganic/ethyl mercury. These null findings could reflect limited exposure to these particular metals in typical tobacco products, or possibly insufficient sample sizes, especially for the e-cigarette-only subset, to detect subtle differences. Additionally, the relatively short duration of use in some participants could mean that certain heavy metals have not yet accumulated to a measurable extent. This can be explained by the fact that fish consumption is the largest source of mercury exposure to US citizens, which further indicates that the mercury levels examined in this data likely originate from other sources [32].

Notably, although our Introduction cites recent data suggesting that around 30% of young adults may experiment with e-cigarettes, our study identified only about 10% of participants as exclusive e-cig users. This lower proportion primarily reflects the strict classification criteria we employed, which required no other tobacco use or secondhand smoke exposure in the previous five days. While this approach ensures clear “exclusive use” groups for biomarker comparison, it also narrows the e-cigarette-only group and should not be interpreted as a national prevalence estimate.

### 4.1. Potential Mechanisms Underlying Observed Effects

The mechanisms that link combustible tobacco smoking to higher inflammatory markers, altered blood counts, and elevated heavy metals are multifaceted. Chronic smoking introduces thousands of chemicals, including reactive oxygen species (ROS), that promote cellular injury, oxidative stress, and systemic inflammation [33]. Over time, these insults elevate WBC counts and inflammatory cytokines while also stimulating the bone marrow to increase red blood cell mass to offset diminished tissue oxygenation. Furthermore, tobacco plants naturally accumulate metals like cadmium and lead from the soil, resulting in inhalation and systemic absorption among those who use combustible tobacco [34].

For e-cigarettes, nicotine is delivered via aerosol generated from a heated e-liquid solution. While these solutions often contain fewer of the combustion byproducts found in cigarettes, heating coils (which can be made of metals such as nickel, chromium, or other alloys) and flavoring chemicals can introduce novel exposures. Nonetheless, it appears from these data that e-cigarette users had substantially lower cadmium and lead levels compared to combustible tobacco users, suggesting these metals may be less prominent in e-cigarette aerosols. Whether other metals (e.g., nickel, chromium) or unmeasured toxins might be elevated was not assessed in the present analysis.

### 4.2. Strengths and Limitations

Strengths of this study include the use of a large, nationally representative NHANES sample, robust measures of tobacco use that distinguish exclusive e-cigarette or combustible tobacco use, and detailed laboratory assessments of inflammation and toxic metals. Additionally, the rigorous survey-weighting methodology ensures that the findings are generalizable to the broader U.S. young adult population, a demographic widely recognized as critical to shaping future tobacco use trends.

However, several limitations must be acknowledged. First, this cross-sectional design prevents determining causality or directionality, making it unclear whether tobacco exposure preceded the observed biomarker changes. Second, reliance on self-reported tobacco use, even with strict classification criteria, may introduce recall bias or intentional underreporting. Third, although NHANES includes questions about tobacco use, our classification relied on whether participants reported no product use in the previous five days, which may not capture more nuanced patterns of consumption. The 5-day criterion means someone who smoked a single cigarette in that period was grouped with heavier smokers, and dual users with slightly different timing of product usage could have been excluded. Hence, the generalizability of the exclusive-user findings to broader real-world usage (including dual or intermittent use) is limited. A more nuanced measure (e.g., daily diaries, 30-day consumption) was unavailable in NHANES for this age group. Fourth, while NHANES does offer dietary data (e.g., in the Dietary Module and Diet Behavior & Nutrition questionnaires) and basic occupational information (e.g., job type and work hours), we did not include these variables in the present analysis. Consequently, residual confounding remains possible, as factors such as specific dietary patterns and detailed occupational exposures could still influence the observed associations. Fifth, the relatively small size of the e-cigarette-only group may limit statistical power to detect subtle differences. Finally, NHANES only measures specific metals (lead, cadmium, mercury, selenium, and manganese) in the 18–30 age group, leaving other relevant metals (such as nickel and chromium) unassessed.

### 4.3. Clinical and Public Health Implications

From a public health perspective, these findings reinforce the well-documented risks of combustible tobacco. Higher inflammatory markers, heavier metal burden, and potential for long-term harm underscore the need for continued prevention and cessation efforts. Encouragingly, the comparatively lower heavy metal exposures in exclusive e-cigarette users suggests that complete substitution of combustible tobacco with e-cigarettes could reduce some toxic exposures; however, this does not imply that e-cigarettes are unequivocally safe. Inflammation-related changes might take time to manifest, and additional metals or chemicals (not measured here) could pose other risks. Moreover, the fact that many young e-cigarette users have no prior history of combustible tobacco use raises concerns about novel nicotine dependence [7,12].

Policymakers and healthcare providers should remain cautious in framing e-cigarettes as “risk-free” alternatives, especially for young adults. Targeted health education emphasizing that e-cigarettes are not harmless, and that complete avoidance of all tobacco products remains the safest course, could mitigate misunderstanding [35]. Given the growing popularity of e-cigarettes among younger cohorts, continued surveillance of evolving product designs (e.g., pod-based systems, disposable vapes) and updated analyses of their toxicological profiles are imperative.

### 4.4. Future Directions

Several avenues for further investigation emerge from these findings. First, longitudinal studies are needed to clarify whether the observed biomarker differences—especially inflammatory markers and heavy metal exposures in exclusively e-cigarette users—persist or worsen over time, and whether e-cigarette users might eventually develop further abnormalities. Second, because e-cigarette products vary widely in coil composition, flavor chemicals, and nicotine concentration, future research should differentiate between device types and usage patterns to identify specific components that may drive changes in monocyte counts, trace metal levels, or other biomarkers. Third, given that unassessed metals (e.g., nickel, chromium) are present in certain coils and have been detected in aerosol emissions, incorporating a broader panel of heavy metal tests could yield a more comprehensive toxicological profile of e-cigarette use. Fourth, investigations into the mechanisms underlying reduced selenium among e-cigarette users may clarify the interplay between aerosol constituents and antioxidant pathways. Finally, as many young adults adopt e-cigarettes without a history of combustible tobacco use, long-term, prospective cohort designs will be essential to determine whether early e-cigarette initiation predisposes users to distinct cardiovascular, respiratory, or metabolic risks over the lifespan.

## 5. Conclusions

In this nationally representative sample of U.S. young adults, exclusive cigarette smoking was consistently associated with significant alterations in immune cell counts, inflammation markers, and metal burdens in blood. By comparison, exclusive e-cigarette use showed fewer deviations from non-tobacco users, although some subtle differences emerged. Higher mean corpuscular hemoglobin concentration was also observed in e-cigarette-only users relative to non-tobacco users. These findings underscore that e-cigarettes may confer lower risk than combustible tobacco with respect to certain toxic metals and some inflammatory indices, although they should not be presumed harmless. Ongoing, rigorous research on the long-term health impacts of exclusive e-cigarette use, especially among a generation that increasingly views vaping as benign, remains essential to inform evidence-based tobacco control and clinical recommendations.

## Figures and Tables

**Table 1:** Characteristics of study participants stratified by tobacco use status.

Characteristic	No Tobacco (SE)	E-Cigs Only (SE)	Combustible Only (SE)
N (unweighted)	229	74	453
Male	0.6087 (0.0354)	0.5223 (0.0562)	0.5751 (0.0275)
Female	0.3913 (0.0354)	0.4777 (0.0562)	0.4249 (0.0275)
Age	25.01 (0.20)	22.21 (0.41)	23.67 (0.17)
BMI	27.98 (0.58)	26.89 (1.52)	27.10 (0.36)
Non-Hispanic White	0.6326 (0.0339)	0.4817 (0.0795)	0.6254 (0.0351)
Non-Hispanic Black	0.0575 (0.0127)	0.0444 (0.0207)	0.1433 (0.0240)
Mexican American	0.1215 (0.0193)	0.1974 (0.0855)	0.0866 (0.0173)
Other Hispanic	0.0893 (0.0242)	0.1595 (0.0599)	0.0727 (0.0140)
Non-Hispanic Asian	0.0563 (0.0117)	0.0082 (0.0066)	0.0204 (0.0048)
Other/Multi-Racial	0.0429 (0.0137)	0.1088 (0.0461)	0.0517 (0.0099)

Mean (SE) is shown for continuous variables (age, BMI), and proportion (SE) for categorical variables (sex and race/ethnicity). Unweighted sample sizes (N) are presented for each tobacco use category (no tobacco use, e-cigarette use only, and combustible use only). Note: The relatively high proportion of “Combustible Only” users (59.9%) in the final sample arises from strict criteria requiring no other tobacco forms and no secondhand smoke exposure. Thus, these proportions should not be interpreted as representative of the general U.S. population.

**Table 2:** Sample size, weighted mean (±SE) of laboratory markers by tobacco group.

Biomarker	No Tobacco *Sample Size* Mean (SE)	E-Cigs Only *Sample Size* Mean (SE)	Combustible Only *Sample Size* Mean (SE)
White Blood Cell (10^3^/µL)	*215* 7.4195 (0.1696)	*68* 7.1234 (0.3150)	*430* 8.0274 (0.1540)
Red Blood Cell (10^6^/µL)	*215* 4.8003 (0.0480)	*68* 4.6952 (0.0451)	*430* 4.8391 (0.0320)
Hemoglobin (g/dL)	*215* 14.3305 (0.1170)	*68* 14.1140 (0.1900)	*430* 14.5899 (0.0938)
Hematocrit (%)	*215* 42.2712 (0.3455)	*68* 41.3161 (0.4665)	*430* 43.1376 (0.2589)
Mean Corpuscular Volume (fL)	*215* 88.3101 (0.3912)	*68* 88.1649 (0.5737)	*430* 89.3539 (0.2987)
Mean Corpuscular Hemoglobin (pg)	*215* 29.9416 (0.1765)	*68* 30.1149 (0.2958)	*430* 30.2124 (0.1190)
Mean Corpuscular Hgb Conc. (g/dL)	*215* 29.9416 (0.1765)	*68* 30.1149 (0.2958)	*430* 30.2124 (0.1190)
Red Cell Distribution Width (%)	*215* 13.2920 (0.0891)	*68* 13.1593 (0.0946)	*430* 13.4001 (0.0654)
Platelet Count (10^3^/µL)	*215* 244.2154 (4.3853)	*68* 244.4208 (9.6521)	*430* 246.9329 (3.8794)
Mean Platelet Volume (fL)	*215* 8.3474 (0.0687)	*68* 8.5488 (0.1363)	*430* 8.4907 (0.0476)
Nucleated RBC (10^3^/µL)	*48* 0.0835 (0.0187)	*56* 0.0817 (0.0111)	*29* 0.0823 (0.0185)
Lymphocyte Number (10^3^/µL)	*214* 2.3225 (0.0601)	*68* 2.0758 (0.1037)	*428* 2.4391 (0.0591)
Monocyte Number (10^3^/µL)	*214* 0.5925 (0.0145)	*68* 0.5125 (0.0280)	*428* 0.6308 (0.0116)
Neutrophil Number (10^3^/µL)	*214* 4.2470 (0.1271)	*68* 4.3031 (0.2134)	*428* 4.6938 (0.1094)
Eosinophil Number (10^3^/µL)	*214* 0.2184 (0.0164)	*68* 0.1840 (0.0153)	*428* 0.2096 (0.0105)
Basophil Number (10^3^/µL)	*214* 0.0439 (0.0039)	*68* 0.0419 (0.0064)	*428* 0.0488 (0.0037)
C-Reactive Protein (mg/L)	*124* 2.9683 (0.5362)	*60* 2.1041 (0.5001)	*204* 3.4521 (0.5830)
Lead (µg/dL)	*134* 0.7658 (0.0367)	*60* 0.6583 (0.1267)	*241* 0.9057 (0.0446)
Cadmium (µg/L)	*134* 0.2664 (0.0208)	*60* 0.1377 (0.0085)	*241* 0.7497 (0.0468)
Mercury Total (µg/L)	*134* 1.1478 (0.2644)	*60* 0.6147 (0.1293)	*241* 0.8279 (0.0841)
Selenium (µg/L)	*134* 193.0755 (2.6681)	*60* 177.6772 (2.5559)	*241* 193.3342 (2.2452)
Manganese (µg/L)	*134* 9.9063 (0.3360)	*60* 9.6255 (0.4379)	*241* 9.5249 (0.2024)
Mercury (Inorganic) (ug/dL)	*134* 0.1327 (0.0152)	*60* 0.1021 (0.0185)	*241* 0.1101 (0.0139)
Mercury (Ethyl) (ug/dL)	*134* 0.0276 (0.0026)	*60* 0.0250 (0.0000)	*241* 0.0262 (0.0012)
Mercury (Methyl) (ug/dL)	*134* 1.0433 (0.2547)	*60* 0.5093 (0.1257)	*241* 0.7103 (0.0805)

**Table 3:** Survey-weighted linear regression (CBC and inflammation).

Biomarker	User Status	β	Lower_ci	Upper_ci	*p* Value
White Blood Cell (10^3^/µL)	E-Cigs Only	−0.156	−0.819	0.507	0.636
White Blood Cell (10^3^/µL)	Combustible Only	0.829	0.467	1.192	<0.001
Red Blood Cell (10^6^/µL)	E-Cigs Only	−0.043	−0.163	0.077	0.476
Red Blood Cell (10^6^/µL)	Combustible Only	0.072	−0.030	0.173	0.163
Hemoglobin (g/dL)	E-Cigs Only	0.005	−0.329	0.340	0.975
Hemoglobin (g/dL)	Combustible Only	0.418	0.151	0.685	0.003
Hematocrit (%)	E-Cigs Only	−0.357	−1.263	0.549	0.428
Hematocrit (%)	Combustible Only	1.262	0.506	2.019	0.002
Mean Corpuscular Volume (fL)	E-Cigs Only	−0.08	−1.279	1.119	0.893
Mean Corpuscular Volume (fL)	Combustible Only	1.240	0.294	2.186	0.012
Mean Corpuscular Hemoglobin (pg)	E-Cigs Only	0.237	−0.306	0.780	0.381
Mean Corpuscular Hemoglobin (pg)	Combustible Only	0.390	−0.045	0.824	0.077
Mean Corpuscular Hgb Conc. (g/dL)	E-Cigs Only	0.305	0.070	0.539	0.012
Mean Corpuscular Hgb Conc. (g/dL)	Combustible Only	−0.021	−0.215	0.174	0.83
Red Cell Distribution Width (%)	E-Cigs Only	−0.163	−0.416	0.089	0.197
Red Cell Distribution Width (%)	Combustible Only	0.062	−0.138	0.261	0.535
Platelet Count (10^3^/µL)	E-Cigs Only	1.064	−17.933	20.061	0.910
Platelet Count (10^3^/µL)	Combustible Only	4.478	−5.386	14.343	0.363
Mean Platelet Volume (fL)	E-Cigs Only	0.108	−0.250	0.466	0.544
Mean Platelet Volume (fL)	Combustible Only	0.122	−0.045	0.290	0.148
Nucleated RBC (10^3^/µL)	E-Cigs Only	0.003	−0.055	0.061	0.893
Nucleated RBC (10^3^/µL)	Combustible Only	−0.003	−0.071	0.065	0.901
Lymphocyte Number (10^3^/µL)	E-Cigs Only	−0.193	−0.411	0.025	0.081
Lymphocyte Number (10^3^/µL)	Combustible Only	0.152	0.002	0.303	0.048
Monocyte Number (10^3^/µL)	E-Cigs Only	−0.066	−0.137	0.005	0.066
Monocyte Number (10^3^/µL)	Combustible Only	0.046	0.011	0.081	0.011
Neutrophil Number (10^3^/µL)	E-Cigs Only	0.119	−0.407	0.646	0.648
Neutrophil Number (10^3^/µL)	Combustible Only	0.620	0.299	0.940	<0.001
Eosinophil Number (10^3^/µL)	E-Cigs Only	−0.025	−0.071	0.021	0.277
Eosinophil Number (10^3^/µL)	Combustible Only	−0.004	−0.052	0.045	0.876
Basophil Number (10^3^/µL)	E-Cigs Only	0.003	−0.012	0.018	0.692
Basophil Number (10^3^/µL)	Combustible Only	0.008	−0.004	0.020	0.190
C-Reactive Protein (mg/L)	E-Cigs Only	−0.061	−0.565	0.444	0.804
C-Reactive Protein (mg/L)	Combustible Only	0.080	−0.212	0.371	0.574

Estimates (±95% CI) of CBC measures and inflammation markers among participants aged 18–30, comparing e-cigarette-only and combustible tobacco-only users to non-users. β columns show the difference (untransformed or logged, as applicable) relative to the no-tobacco reference group, and “*p*-value” indicates statistical significance at α = 0.05.

**Table 4:** Survey-weighted linear regression (metals).

Biomarker	User Status	β	Lower_ci	Upper_ci	*p*-Value
Lead (µg/dL)	E-Cigs Only	−0.162	−0.454	0.129	0.265
Lead (µg/dL)	Combustible Only	0.184	0.058	0.311	0.005
Cadmium (µg/L)	E-Cigs Only	−0.513	−0.793	−0.233	0.001
Cadmium (µg/L)	Combustible Only	0.954	0.733	1.176	<0.001
Mercury Total (µg/L)	E-Cigs Only	−0.165	−0.602	0.271	0.446
Mercury Total (µg/L)	Combustible Only	−0.025	−0.324	0.274	0.865
Selenium (µg/L)	E-Cigs Only	−0.08	−0.127	−0.034	0.001
Selenium (µg/L)	Combustible Only	0.002	−0.029	0.034	0.877
Manganese (µg/L)	E-Cigs Only	−0.044	−0.173	0.085	0.489
Manganese (µg/L)	Combustible Only	−0.015	−0.077	0.048	0.641
Mercury (Inorganic) (ug/dL)	E-Cigs Only	−0.16	−0.364	0.044	0.119
Mercury (Inorganic) (ug/dL)	Combustible Only	−0.109	−0.259	0.041	0.148
Mercury (Ethyl) (ug/dL)	E-Cigs Only	−0.017	−0.06	0.027	0.445
Mercury (Ethyl) (ug/dL)	Combustible Only	−0.011	−0.08	0.058	0.743
Mercury (Methyl) (ug/dL)	E-Cigs Only	−0.393	−0.784	−0.001	0.049
Mercury (Methyl) (ug/dL)	Combustible Only	−0.102	−0.357	0.154	0.424

Estimates (±95% CI) of blood heavy metals among participants aged 18–30, comparing e-cigarette only and combustible tobacco only users to non-users. β columns show the logged difference relative to the no-tobacco reference group, and “*p*-value” indicates statistical significance at α = 0.05.

## Data Availability

The NHANES data used in this study (2013–2023) are publicly available at https://wwwn.cdc.gov/nchs/nhanes (accessed on 15 March 2025).

## References

[1] Centers for Disease Control and Prevention (2024). Tobacco Product Use among Adults—United States, 2022. https://www.cdc.gov/tobacco/media/pdfs/2024/09/cdc-osh-ncis-data-report-508.pdf.

[2] Erhabor J., Boakye E., Obisesan O., Osei A.D., Tasdighi E., Mirbolouk H., DeFilippis A.P., Stokes A.C., Hirsch G.A., Benjamin E.J. (2023). E-Cigarette Use Among US Adults in the 2021 Behavioral Risk Factor Surveillance System Survey. JAMA Netw. Open.

[3] Boakye E., Osuji N., Erhabor J., Obisesan O., Osei A.D., Mirbolouk M., Stokes A.C., Dzaye O., El Shahawy O., Hirsch G.A. (2022). Assessment of Patterns in e-Cigarette Use Among Adults in the US, 2017–2020. JAMA Netw. Open.

[4] Chen-Sankey J.C., Unger J.B., Bansal-Travers M., Niederdeppe J., Bernat E., Choi K. (2019). E-cigarette Marketing Exposure and Subsequent Experimentation Among Youth and Young Adults. Pediatrics.

[5] McCausland K., Maycock B., Leaver T., Jancey J. (2019). The Messages Presented in Electronic Cigarette–Related Social Media Promotions and Discussion: Scoping Review. J. Med. Internet Res..

[6] Balfour D.J.K., Benowitz N.L., Colby S.M., Hatsukami D.K., Lando H.A., Leischow S.J., Lerman C., Mermelstein R.J., Niaura R., Perkins K.A. (2021). Balancing Consideration of the Risks and Benefits of E-Cigarettes. Am. J. Public Health.

[7] Lin C., Gaiha S.M., Halpern-Felsher B. (2022). Nicotine Dependence from Different E-Cigarette Devices and Combustible Cigarettes among US Adolescent and Young Adult Users. Int. J. Environ. Res. Public Health.

[8] Komiyama M., Swati M., Yamakage H., Morimoto T., Hasegawa K. (2023). Effect of smoking initiation age on nicotine dependence. Eur. Heart J..

[9] Ali F.R.M., Agaku I.T., Sharapova S.R., Reimels E.A., Homa D.M. (2020). Onset of Regular Smoking Before Age 21 and Subsequent Nicotine Dependence and Cessation Behavior Among US Adult Smokers. Prev. Chronic Dis..

[10] Sharapova S., Reyes-Guzman C., Singh T., Phillips E., Marynak K.L., Agaku I. (2020). Age of tobacco use initiation and association with current use and nicotine dependence among US middle and high school students, 2014–2016. Tob. Control.

[11] Pierce J.P., Luo M., McMenamin S.B., Stone M.D., Leas E.C., Strong D., Shi Y., Kealey S., Benmarhnia T., Messer K. (2023). Declines in cigarette smoking among US adolescents and young adults: Indications of independence from e-cigarette vaping surge. Tob. Control.

[12] Rodu B., Plurphanswat N. (2024). Joint smoking–vaping prevalence rates among American youth and young adults 2011–2022. Harm Reduct. J..

[13] Bandi P., Cahn Z., Goding Sauer A., Douglas C.E., Drope J., Jemal A., Fedewa S.A. (2021). Trends in E-Cigarette Use by Age Group and Combustible Cigarette Smoking Histories, U.S. Adults, 2014–2018. Am. J. Prev. Med..

[14] Pedersen K.M., Çolak Y., Ellervik C., Hasselbalch H.C., Bojesen S.E., Nordestgaard B.G. (2019). Smoking and Increased White and Red Blood Cells. Arterioscler. Thromb. Vasc. Biol..

[15] Das I. (1985). Raised C-reactive protein levels in serum from smokers. Clin. Chim. Acta.

[16] O’Loughlin J., Lambert M., Karp I., McGrath J., Gray-Donald K., Barnett T., Delvin E., Levy E., Paradis G. (2008). Association between cigarette smoking and C-reactive protein in a representative, population-based sample of adolescents. Nicotine Tob. Res..

[17] Koh D.-H., Choi S., Park J.-H., Lee S.-G., Kim H.-C., Kim I., Park D.-U. (2024). Evaluation on the Sex-Specific Association Between Cigarette Smoke Exposure and Inflammation Markers—C-Reactive Protein and White Blood Cell Count. Nicotine Tob. Res..

[18] Shakeri M.T., Nezami H., Nakhaee S., Aaseth J., Mehrpour O. (2021). Assessing Heavy Metal Burden Among Cigarette Smokers and Non-smoking Individuals in Iran: Cluster Analysis and Principal Component Analysis. Biol. Trace Elem. Res..

[19] Goniewicz M.L., Smith D.M., Edwards K.C., Blount B.C., Caldwell K.L., Feng J., Wang L., Christensen C., Ambrose B., Borek N. (2018). Comparison of Nicotine and Toxicant Exposure in Users of Electronic Cigarettes and Combustible Cigarettes. JAMA Netw. Open.

[20] Simovic T., Matheson C., Rodgers A., Baig F., Breland A., Caroline Cobb O., Nana-Sinkam P. (2024). Immune Cell Profile in Young Adults who Regularly Use E-cigarettes and the Role of Nicotine. Physiology.

[21] Chatterjee S., Tao J.-Q., Johncola A., Guo W., Caporale A., Langham M.C., Wehrli F.W. (2019). Acute exposure to e-cigarettes causes inflammation and pulmonary endothelial oxidative stress in nonsmoking, healthy young subjects. Am. J. Physiol. Lung Cell. Mol. Physiol..

[22] Banks E., Yazidjoglou A., Joshy G. (2023). Electronic cigarettes and health outcomes: Epidemiological and public health challenges. Int. J. Epidemiol..

[23] Marques P., Piqueras L., Sanz M.-J. (2021). An updated overview of e-cigarette impact on human health. Respir. Res..

[24] Centers for Disease Control and Prevention (CDC) (2025). National Health and Nutrition Examination Survey. https://www.cdc.gov/nchs/nhanes/about.

[25] (2023). R Core Team. https://www.R-project.org.

[26] Lumley T. (2004). Analysis of Complex Survey Samples. J. Stat. Softw..

[27] Phillips B., Titz B., Kogel U., Sharma D., Leroy P., Xiang Y., Vuillaume G., Lebrun S., Sciuscio D., Ho J. (2017). Toxicity of the main electronic cigarette components, propylene glycol, glycerin, and nicotine, in Sprague-Dawley rats in a 90-day OECD inhalation study complemented by molecular endpoints. Food Chem. Toxicol..

[28] Merecz-Sadowska A., Sitarek P., Zielinska-Blizniewska H., Malinowska K., Zajdel K., Zakonnik L., Zajdel R. (2020). A Summary of In Vitro and In Vivo Studies Evaluating the Impact of E-Cigarette Exposure on Living Organisms and the Environment. Int. J. Mol. Sci..

[29] Prokopowicz A., Sobczak A., Szuła-Chraplewska M., Ochota P., Kośmider L. (2019). Exposure to Cadmium and Lead in Cigarette Smokers Who Switched to Electronic Cigarettes. Nicotine Tob. Res..

[30] Badea M., Luzardo O.P., González-Antuña A., Zumbado M., Rogozea L., Floroian L., Alexandrescu D., Moga M., Gaman L., Radoi M. (2018). Body burden of toxic metals and rare earth elements in non-smokers, cigarette smokers and electronic cigarette users. Environ. Res..

[31] Ellis N.I., Lloyd B., Lloyd R.S., Clayton B.E. (1984). Selenium and vitamin E in relation to risk factors for coronary heart disease. J. Clin. Pathol..

[32] Martinez-Morata I., Sobel M., Tellez-Plaza M., Navas-Acien A., Howe C.G., Sanchez T.R. (2023). A State-of-the-Science Review on Metal Biomarkers. Curr. Environ. Health Rep..

[33] Caliri A.W., Tommasi S., Besaratinia A. (2021). Relationships among smoking, oxidative stress, inflammation, macromolecular damage, and cancer. Mutat. Res./Rev. Mutat. Res..

[34] Liu H., Wang H., Ma Y., Wang H., Shi Y. (2016). Role of transpiration and metabolism in translocation and accumulation of cadmium in tobacco plants (*Nicotiana tabacum* L.). Chemosphere.

[35] Jayawardene W., Kumbalatara C., Dufner M., Tran P., Redner R. (2025). Vaping Cessation Attempts and Intentions Among Undergraduate Students: Insights into Interventions. J. Coll. Stud. Ment. Health.

